# Removal and Oxidation of As(III) from Water Using Iron Oxide Coated CTAB as Adsorbent

**DOI:** 10.3390/polym12081687

**Published:** 2020-07-29

**Authors:** Daniela Predoi, Simona Liliana Iconaru, Mihai Valentin Predoi, Mikael Motelica-Heino

**Affiliations:** 1 National Institute of Materials Physics, Atomistilor Street, No. 405A, P.O. Box MG 07, 077125 Magurele, Romania; simonaiconaru@gmail.com; 2 Department of Mechanics, University Politehnica of Bucharest, BN 002, 313 Splaiul Independentei, Sector 6, 060042 Bucharest, Romania; predoi@gmail.com; 3 ISTO, UMR 7327 CNRS Université d’Orléans, 1A rue de la Férollerie, 45071 Orléans, France; mikael.motelica@univ-orleans.fr

**Keywords:** adsorption, arsenic, CTAB coated iron oxide, removal, ultrasound measurements

## Abstract

Iron oxides such as magnetite and maghemite coated with cetyltrimethylammonium bromide (CTAB) are very promising materials for wastewater treatment because iron oxide can be easily separated from solutions using the magnetic separation procedure Iron oxide (IO) coated CTAB was synthesized by an adapted co-precipitation method. In the present study, the IO-CTAB was used for removing arsenic from water for the first time. In the present study, the performance of iron oxide coated CTAB biocomposites as an adsorbent for arsenic removal from aqueous solutions was examined. X-ray diffraction (XRD) analysis and the results revealed a cubic phase Fd-3 m of Fe_3_O_4_ with lattice a = 8.40 Å and average crystal size equal to 17.26 ± 3 nm. The mean particle size calculated from transmission electron microscopy (TEM) was 19.86 ±1.7 nm. The results of the adsorption batch experiments and the data determined using the Langmuir and Freundlich models emphasized that IO-CTAB nanoparticles were favorable for the adsorption of As(III) ions from aqueous solutions. Ultrasound measurements have shown that IO-CTAB is a cost-effective biocomposite for removing arsenic from contaminated solutions. Moreover, x-ray photoelectron spectroscopy (XPS) has shown that during the process of arsenic absorption, there is oxidation from As(III) to As(V), which leads to a decrease in toxicity during this process. The results of the cytotoxic assays confirmed that the IO-CTAB nanoparticles did not induce any morphological changes in the HeLa cells and did not affect their proliferation after 24 h of incubation.

## 1. Introduction

In the last decades due to rapid technological development, heavily industrialized countries are severely affected due to the continuous and rapid expansion of contaminated areas worldwide. Thus, recently, the design and manufacture of materials with new specific properties that could be used successfully in remedying the environment has become a priority among researchers working in various scientific fields. An increased interest in the development of novel materials for environmental applications has been given to nanoscale materials due to their specific properties caused by the increase of the surface due to the decrease of the volume/surface ratio. During the years, the effectiveness of mineral-based decontamination materials such as carbonate, lime, and phosphates that are used to reduce metal toxicity has been extensively studied [1].

The most common studied mineral materials include silicates, aluminosilicates or clay minerals, sulfates, zero-valence iron, and iron hydroxides [2]. Nanoscale materials have special physico-chemical properties compared to those of micro and/or macroscopic materials, which lead to an increase in their efficiency in environmental remediation processes.

The specific properties of nanometric materials that could lead to their capacity of removing pollutants from contaminated waters and soils are their large specific area, their ability to be functionalized through surface bonds that increase their affinity for various pollutants, and their ability to be used as selective ligands for certain toxic elements and organic/inorganic substances dissolved in aqueous media. Adsorption is the most frequently used mechanism for the removal of organic and inorganic impurities from contaminated wastewaters, inland, or ocean waters as well as from soils. The efficiency of conventional adsorbents is usually limited due to the specific surface area, lack of selectivity, etc., while nano-adsorbents offer a significant improvement due to the increase of their specific surface area and porosity [3].

The mechanism and adsorption kinetics for different adsorbents depend on the chemical nature of the materials and the experimental conditions such as the pH of the solution, the adsorbent/pollutant ratio, and the system temperature, etc. [4]. In the past decades, arsenic was recognized as a major contaminant of drinking water. In West Bengal and Bangladesh in the 1990s, about 20 years after the construction of tubular fountains that gave the population access to drinking water, it was discovered that arsenic contamination posed a serious health threat to the population. It has been estimated that over 50 million people in Bangladesh consume groundwater contaminated with arsenic [5].

Arsenic poisoning can cause serious symptoms such as skin lesions, hyperkeratosis, melanosis, skin cancer, and cancer of other organs. Following the recognition of the first cases of arsenic poisoning in the population of West Bengal and Bangladesh, similar cases have been identified worldwide. In his study of technologies for removing arsenic from contaminated environments, Krzysztof P. Kowalski [5] stated that more than 100 million people worldwide are exposed to drinking water contaminated with arsenic. In this context, arsenic contamination of continental and ocean waters has been one of the most important research topics, and the results of the studies conducted has allowed the World Health Organization (WHO) to set the level of arsenic accepted in drinking water at 10 µg/L [6].

Studies have emphasized that drinking water contaminated with arsenic causes serious health problems such as cancer and other life treating illnesses. Long-term solutions are needed in the affected areas to provide the population with a source of uncontaminated drinking water. However, in the near future, there is no improvement in the level of contamination of drinking water sources. In this context, the development of new materials with biocompatible properties to remove arsenic from the groundwater is a major area of global interest [7,8,9]. Recently, the increasing demand for water consumption and industrial support, together with the harsh environmental legislation, has stimulated the development of new materials and methods for the treatment of arsenic contaminated aqueous solutions.

Research made in the material sciences for environmental applications related to the synthesis and modification of nanoparticles (NPs) has had a great impact on material engineering and surface science applications due to the unique chemical and physical properties of materials at a nanometric scale compared to bulk materials. In recent years, the interest in the efficient synthesis of magnetic iron oxide nanoparticles and their functionalization has increased considerably due to their wide range in various applications as well as their applications for metal ion removal from aqueous solutions [10,11,12,13,14,15,16,17,18]. Previous studies have reported that different iron oxide species such as Fe_3_O_4_, α-Fe_2_O_3_, and ɤ-Fe_2_O_3_ that interact with As had the ability to remove both As(III) and As(V) from water [15,19]. Goethite has been reported to be able to catalyze the oxidation of As(III) to As(V), thereby reducing the mobility of As [20], while other studies [21] have also reported that iron oxide nanoparticles can not only stabilize As, but could also change As speciation.

In water treatment technologies, adsorption processes are usually involved, and the materials used as adsorbents should possess specific features such as small-sized particles and a large specific surface area. Iron oxide nanoparticles with a highly specific surface area have been investigated as low-cost adsorbents with a high adsorption capacity for metal ions, which could also be conveniently recovered by magnetic separation after the decontamination process [10,11,12,13,14,15,16,17,18,19,20,21]. Therefore, this study focused on the development of iron oxide nanoparticles coated with cetyltrimethylammonium bromide (CTAB) nanoparticles for environmental applications.

According to the literature, in this study, we present for the first time the results concerning the removal and oxidation of As(III) from water using iron oxide coated CTAB as an adsorbent. The efficiency of the IO-CTAB biocomposite in removing arsenic from contaminated water was evaluated by three complementary methods such as adsorption kinetics, ultrasound measurements, and X-ray photoelectron spectroscopy (XPS). On the other hand, ultrasound studies have shown a behavior similar to that of double distilled water for depolluted water. Moreover, cell viability tests were performed on the IO-CTAB biocomposite used in the removal of arsenic, but also on contaminated and decontaminated waters.

## 2. Materials and Methods

### 2.1. Sample Preparation

#### 2.1.1. Materials

Cetyltrimethylammonium bromide ([(C_16_H_33_)N(CH_3_)_3_]Br; CTAB), ferrous chloride tetrahydrate (FeCl_2_⋅4H_2_O), ferric chloride hexahydrate (FeCl_3_⋅6H_2_O), hydrochloric acid (HCl), ammonia (NH_3_), and perchloric acid (HClO_4_) were purchased from Merck (Merck, Kenilworth, NJ, United States). Double distilled water and deionized water were used in the synthesis and for rinsing the clusters.

#### 2.1.2. Synthesis of Cetyltrimethylammonium Bromide (CTAB) Coated Iron Oxide Nanoparticles (IO-CTAB)

The CTAB coated iron oxide (IO-CTAB) nanoparticles were synthesized by coprecipitation by a modified method described previously [22,23,24]. The ferric chloride hexahydratate (FeCl_3_⋅6H_2_O) and ferrous chloride tetrahydrate (FeCl_2_⋅4H_2_O) in 2 M HCl where the ratio of Fe^2^/Fe^3^ was ½ were combined at room temperature and added drop by drop in an aqueous solution under vigorous stirring. The aqueous solution was obtained from 800 mL deionized water, 80 mL NH_3_, and 8 g CTAB. The resulting mixture was stirred for 4 h on a magnetic stirrer. After stirring, the resulting mixture was allowed to settle for 1 h. After the supernatant was removed, 400 mL of ethanol was added and stirred for 1 h. The final magnetic precipitate was separated by centrifugation (10,000 rpm), after which it was washed in deionized water. The precipitate was washed four times. The final precipitate was dried in an oven at 80 °C. The resulting powder was analyzed by different techniques and used in the removal of As from contaminated water.

### 2.2. Characterization

#### 2.2.1. Characterization of CTAB Coated Iron Oxide Aqueous Magnetic Fluids by X-Ray Diffraction

X-ray diffraction patterns for the IO-CTAB composite powder were recorded using a Bruker D8 Advance diffractometer, with nickel filtered Cu K_α_ (λ = 1.5418 Å) radiation, and a high efficiency one-dimensional detector Lynx Eye type (Bruker, Billerica, MA, USA) operated in integration mode. The CuK_α_ wavelength was λ = 1.5418 Å. The average crystal size were assessed using the Scherrer formula [25]:

d = Kλ/β cos θ (1)

where d is the crystal size; K is the Scherrer constant (0.9); λ is the x-ray wavelength of radiation for Cu Kα (1.5418 Å); β is the full-width at half maximum (FWHM) of a diffraction peak measured at 2θ; and θ is the diffraction angle.

#### 2.2.2. Characterization of CTAB Coated Iron Oxide Aqueous Magnetic Fluids by Transmission Electron Microscopy and Scanning Electron Microscopy

The shape and size of IO-CTAB nanoparticles were assessed using a transmission electron microscope (TEM), JEOL 100CX (JEOL, Peabody, MA, USA). The IO-CTAB powder was dispersed in alcohol and mixed. A drop of the suspension was dropped onto a carbon coated copper grid dried in air and analyzed. The morphology of the IO-CTAB composite was evaluated by scanning electron microscopy (SEM) using a HITACHI S2600N-type microscope (HITACHI, Tokyo, Japan) equipped with an energy dispersive x-ray attachment EDAX/2001 (Ametek EDAX Inc., Mahwah, NJ, USA). The EDAX/2001 device was useful in identifying the elemental composition of the IO-CTAB composite. 

### 2.3. Batch Adsorption Experiments

Arsenic removal from contaminated aqueous solutions using iron oxide coated with cetyltrimethylammonium bromide nanoparticles (IO-CTAB) was studied by batch adsorption experiments. The experiments were performed at room temperature using silicon tubes with aqueous solutions contaminated with As(III) ions in a concentration range of 0–100 mg/L. The amount of IO-CTAB nanopowders used in the batch experiments as adsorbents was 0.2 g. The contaminated solution pH was adjusted to a value of 5 using a 0.1 M hydrochloric acid (HCl) solution. The batch adsorption experiments were done maintaining the solution volume at 20 mL while the mixture was stirred using a Mixer SRT1 Roller (Stuart Scientific, Staffordshire, UK) for 24 h. After 24 h, the tubes containing both the contaminated solutions and the 0.2 g of IO-CTAB nanoparticles were centrifuged for 30 min at 10,000 rpm. After they had been centrifuged, the supernatant was filtered, recovered, and analyzed by flame atomic absorption spectrometry (AAS) using a Zeeman HITACHI Z-8100 from Japan Hitachi (Tokyo, Japan). The AAS studies were conducted in triplicate using a constant airflow rate and a wavelength of 193.7 nm according to the operational conditions for arsenic. The removal efficiency of As(III) ions onto IO-CTAB nanoparticles was calculated using the following formula:

(2) $$R\left(\%\right)=\frac{\left(C_{0}-C_{e}\right)}{C_{0}}\cdot 100$$

where Co and Ce are the initial and equilibrium concentrations of As(III) (mg/L) ions.

Furthermore, the kinetics of the adsorption process of arsenic ions on IO-CTAB nanoparticles from the batch experiments were described using the Langmuir and Freundlich adsorption models [26]. Using the Langmuir model, the amount of metal retained per mass unit, also called the adsorption capacity, was calculated. The quantity of As(III) ions adsorbed at equilibrium per unit mass, Q_e_, was determined using the following formula:

(3) $$Q_{e}=\frac{\left(C_{0}-C_{e}\right)}{m}\cdot V$$

where C_0_ (mg/L) is the initial metal ion concentration; C_e_ (mg/L) is the equilibrium concentration of As(III), V (L) is the volume of the solution; and m (g) is the mass of the adsorbent.

The theoretical Langmuir isotherm is often used to describe the adsorption of a dissolved solution from a liquid solution, and is described by the following formula [27,28,29]:

(4) $$Q_{e}=\frac{q_{m}K_{L}C_{e}}{1+K_{L}C_{e}}$$

where q_m_ and K_L_ are the Langmuir constants that define the maximum adsorption capacity and the constant energy associated with the heat of adsorption, respectively.

The Langmuir constants, q_m_ and K_L_ were determined from the graphical representation (C_e_/Q_e_) function of (C_e_) from the linear form of the Langmuir equation:

(5) $$\frac{C_{e}}{Q_{e}}=\frac{1}{\left(q_{m}\cdot K_{L}\right)}+\frac{C_{e}}{q_{m}}$$

In addition, from the experimental from the Langmuir isotherm, the dimensionless separation factor constant R_L_, also called equilibrium parameter, was determined using the following equation [30]:

(6) $$R_{L}=\frac{1}{1+K_{L}C_{0}}$$

where C_0_ (mg/L) is the initial dye concentration and K_L_ (L/mg) is the Langmuir constant related to the energy of adsorption.

The Freundlich isotherm that was used to model the experimental data is also described by the following equation:

(7) Qe=kf·Ce1n

where Q_e_ is the amount of material adsorbed at equilibrium (mg/g); C_e_ is the metal ion concentration at equilibrium (mg/L); and k_f_ [mg/g (mg/L)^−1/n^] and n are Freundlich constants, representing the adsorption capacity and the adsorption intensity of the adsorbent, respectively.

The Freundlich constants, k_f_ and n, were determined using the graphical representation (lnQ_e_) function of (lnC_e_) from the linear form of the Freundlich equation

(8) $$lnQ_{e}=lnk_{f}+\frac{1}{n}lnC_{e}$$

### 2.4. Non-Destructive Ultrasound Studies

In order to evaluate the efficiency of iron oxide coated with CTAB in removing arsenic from polluted waters, ultrasonic studies were performed on the polluted solutions before and after decontamination. Double-distilled water was used as a reference to evaluate the effectiveness of the IO-CTAB composite in removing arsenic. Ultrasound pulses [31] were used to analyze both arsenic-contaminated solutions and solutions obtained after arsenic removal using the IO-CTAB composite.

### 2.5. X-Ray Photoelectron Spectroscopy Studies (XPS)

X-ray photoelectron spectroscopy measurements (XPS) were effectuated using a multimethod SPECS surface analysis system (Specs, Berlin, Germany). The device functions with Al Kα (1486.6 eV) monochromatic radiation. The vacuum in the analysis chamber was p~3 × 10^−9^ torr. X-radiation is emitted by an Al-cathode, the voltage is U = 12.5 kV, and the filament emission current is I = 20 m. To acquire the XPS spectra, an energy window w = 20 eV with resolution R = 20 eV and 400 recording channels was used. The obtained XPS spectra were examined and processed using Spectral Data Processor v2.3 (SDP) [32].

### 2.6. Biological Studies

#### 2.6.1. Quantitative Cell Viability Assay

The cytotoxicity of the iron oxide coated with cetyltrimethylammonium bromide nanoparticles was investigated using the 3-4,5-Dimethylthiazol 2,5-diphenyltetrazolium bromide assay (MTT) in agreement with previous studies [33,34,35]. The *in vitro* cytotoxicity assessment was carried out using HeLa cells. The viability of the cells after being incubated for 24 h with the IO-CTAB nanoparticles as well as contaminated and decontaminated solutions before and after the arsenic removal experiments was quantified by measuring the wavelength at 595 nm using a TECAN spectrophotometer (Tecan GENios, Grödic, Germany). The absorbance from the wells of cells cultured in the absence of the substrate, coatings, and dispersions was used as the 100% viability value. The experiments were done in triplicate and the results presented as ±SD.

#### 2.6.2. Qualitative Evaluation of Cell Viability and Cell Morphology by Fluorescence Microscopy

The morphology of the HeLa cells was also investigated by optical microscopy after 24 h of incubation with the IO-CTAB nanoparticles as well as contaminated and decontaminated solutions before and after the arsenic removal experiments.

### 2.7. Statistical Analyses

All the biological experiments were conducted in triplicate. As a result, all measurements were carried out three times. Data were plotted as mean ± standard deviation (SD) and compared by the Student’s t-test. The P values of less than 0.05 were taken into account from a statistical point of view.

## 3. Results

### 3.1. Characterization of CTAB Coated Iron Oxide Aqueous Magnetic Fluids by X-Ray Diffraction, Transmission Electron Microscopy, and Scanning Electron Microscopy

**Figure 1:**
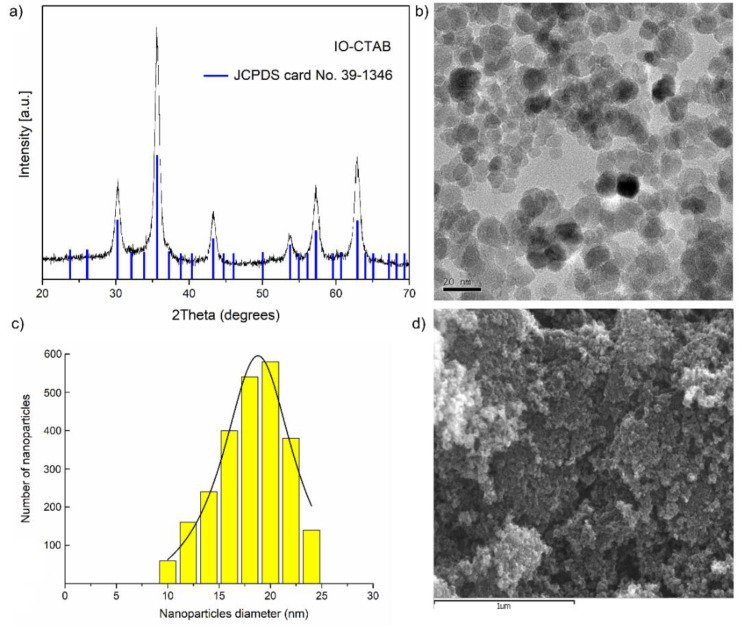
XRD patterns of the prepared IO-CTAB nanoparticles (**a**); TEM image of IO-CTAB nanoparticles (**b**); Size distribution histogram of the synthesized IO-CTAB nanoparticles (**c**); SEM image of IO-CTAB nanoparticles (**d**).

The crystal structure and phase purity of the synthesized CTAB coated iron oxide (IO-CTAB) composite were examined by XRD analysis and the results are presented in Figure 1a. The XRD pattern of IO-CTAB was indexed and resembled to a cubic phase Fd-3m of Fe_3_O_4_ with lattice constant a = 8.40 Å. The calculated value of the lattice parameter of IO-CTAB is in agreement with the previously reported value [36]. The average crystal size of IO-CTAB evaluated using the Scherrer equation was 17.26 ± 3 nm. No obvious XRD peaks corresponding to other phases were found in the XRD model of the analyzed sample, which shows a good purity of the IO-CTAB prepared sample. The shape and morphology of IO-CTAB was examined by TEM and SEM analysis. The TEM image (Figure 1b) demonstrated that the sample was composed of a number of nanoparticles with spherical shapes. The observed IO-CTAB nanoparticles had sizes between 10 and 25 nm. The histogram of particle size distribution was taken after counting about 500 particles from the TEM image (Figure 1c). The mean particle size was 19.86 ±1.7 nm, which is in accordance with the result achieved from the XRD analysis. The SEM image of IO-CTAB showed that the particles were agglomerated with nanometric dimensions and spherical shape (Figure 1d).

### 3.2. Adsorption Kinetics and Isotherms

The efficiency of the IO-CTAB nanoparticles in the removal of arsenic ions from aqueous solutions was investigated using flame atomic absorption spectroscopy. For that purpose, batch adsorption experiments were performed at room temperature. The effect of different pH values (5, 7, and 9) on the adsorption of As(III) by 0.2 g of IO-CTAB nanoparticles after 24 h of contact time was investigated. The results revealed that the pH values had a strong influence on the removal capacity of the IO-CTAB nanoparticles.

**Figure 2:**
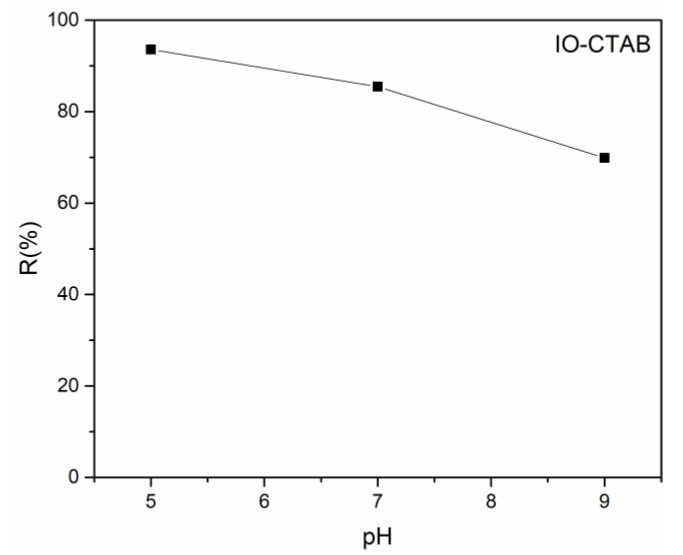
Removal (%) of As(III) from contaminated aqueous solutions using IO-CTAB nanoparticles at different pH values.

The results are presented in Figure 2. The studies emphasize that the best removal efficiency of As(III) from aqueous solutions using IO-CTAB nanoparticles was obtained at pH 5 as the removal efficiency decreased with the increase in the pH value. These results are in good agreement with studies regarding the applications of CTAB modified magnetic nanoparticles for removal of chromium(VI) from contaminated water conducted by Elfeky et al. [37]. The studies reported by Elfeky et al. [37] revealed that the maximum adsorption of Cr(VI) onto Fe_3_O_4_ nanoparticles and Fe_3_O_4_/CTAB was observed at pH 4 after eight hours of contact time. Moreover, their results also showed that and Fe_3_O_4_/CTAB were more efficient than Fe_3_O_4_ in the removal of Cr(VI) from contaminated solutions.

Furthermore, the effect of the initial concentration of As(III) of the solution on the efficiency of IO-CTAB nanoparticles in the removal of arsenic ions from aqueous solutions at pH 5 was investigated using solutions with different concentrations of As(III) in the range of 0–100 mg/L.

**Figure 3:**
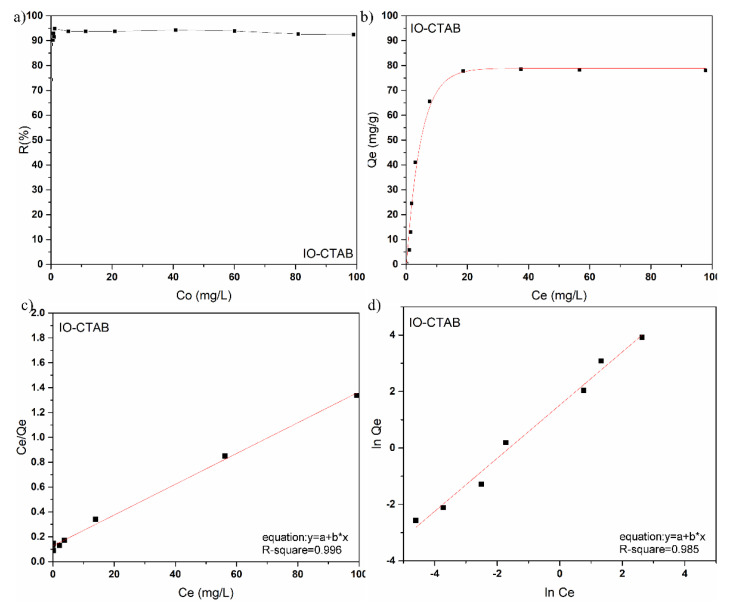
The effect of the initial concentration of As(III) of the solution on the removal (%) of As(III) from contaminated aqueous solutions using IO-CTAB nanoparticles (**a**); graphic representation of the amount of material adsorbed at equilibrium (**b**); Langmuir linearized fits (**c**); Freundlich linearized fits (**d**).

Figure 3a presents the effect of the initial concentration of As(III) on the removal of arsenic ions from aqueous solutions by IO-CTAB nanoparticles. It can be seen that the arsenic removal efficiency depends on the initial concentration of As(III). For an arsenic concentration smaller than 5 mg/L, the removal efficiency of arsenic ions from the contaminated solution of IO-CTAB nanoparticles was between 88% and 90%, thus demonstrating that the adsorbent material (IO-CTAB nanoparticles) had a strong affinity for As(III) ions.

For arsenic concentrations in the range of 10 mg/L to 100 mg/L, it could be seen that the removal efficiency of As(III) ions was approximately 93%.

During the years, the processes of adsorption of metal ions on different materials have been described using several models such as the Langmuir, Freundlich, Temkin, Toth, Hill, Flory-Huggins, and Radke-Prausnitz, etc. [38]. In all of the described models, the kinetic aspect had an important role. In the literature, the adsorption equilibrium was defined as the dynamic equilibrium state, with both adsorption and desorption rates [39]. The models consider the physico-chemical parameters taken together with basic thermodynamic assumptions and could give information on the adsorption mechanisms, surface properties, and adsorbent affinity [40]. The adsorption process of arsenic ions on IO-CTAB nanoparticles from the batch experiments were described using the Langmuir and Freundlich adsorption models [26].

The adsorption isotherms for the arsenic removal by IO-CTAB nanoparticles were obtained by mixing solutions containing different concentrations of As(III) with 0.2 g of IO-CTAB nanoparticles until thermodynamic equilibrium at ambient temperature (T = 25 °C) was achieved. Previously reported studies emphasized that the thermodynamic equilibrium at ambient temperature can be achieved in less than 24 h [13,14,15,16,17,18,19,20,21].

The Langmuir model of adsorption and the Langmuir isotherm was developed for the description of the gas–solid adsorption phase in activated carbon. Since then, it has been used mostly to quantify the efficiency of numerous materials used as adsorbents [39]. The empirical model only refers to monolayer adsorption (in this case, the adsorbed layer has the thickness of a molecule). In addition, in the Langmuir model, the adsorption can only take place at a finite number of localized and defined areas that are identical and equivalent [41,42] and usually the graphical representation of the Langmuir isotherm is characterized by a saturation zone [27].

Figure 3b presents the experimental data and the theoretical Langmuir model when the IO-CTAB nanoparticles were used for the adsorption of As(III) ions from aqueous solutions. The graphical representations of the As(III) adsorbed ions on the mass unit by IO-CTAB nanoparticles (q_e_), depending on the concentration of As(III) remaining in solution (C_e_) are shown in Figure 3b.

Furthermore, in order to have a complex description of the adsorption mechanism, the graphical representation of the C_e_/Q_e_ function of C_e_ was also obtained and is depicted in Figure 3c. The results of the studies showed that at ambient temperature, the R^2^ coefficient calculated from the Langmuir isotherm was equal to 0.996 for IO-CTAB. In good agreement with previous studies [37,43,44], the transformation of the nonlinear isothermal equation into the linear form by a nonlinear method has not raised any problems. The q_m_ and K_L_ Langmuir constants were calculated using the graphical representation of the (C_e_/Q_e_) function of (C_e_). In the last few years, studies have been reported in the literature on the ability of different metal oxides to adsorb metal ions from aqueous solutions. Considering the experimental conditions and the adsorption process parameters such as pH solution, sorbent mass, initial concentration of pollutant material, and the experiment duration, the adsorption capacity of As(III) ions by iron oxide or iron oxide composites was reported between 8 and 66.53 mg/g [11,45,46]. Nonetheless, during the years, extensive research has been conducted with the sole purpose of improving the properties of adsorbents [13,14,15,16,17,18,19,20,21]. Moreover, in their recent studies, Otero-González et al. [13] reported that the estimated maximum adsorption values (q_max_) values obtained in the case of As(III) retention on novel nanostructured iron oxide cryogels were 625 and 588 mg As(III) per g of Fe for the IO gel and the IO/AAm/MBAA cryogel, respectively.

Following the reaction of IO-CTAB with solutions contaminated with As(III) ions, the arsenic ions were removed from the solutions. The Langmuir q_m_ and K_L_ constants, representing the maximum adsorption capacity, and the constant energy associated with the heat of adsorption, respectively, were also determined using the graphical representation of the linear form of the Langmuir equation. The results obtained from the adsorption batch experiments to remove arsenic ions from aqueous solutions using IO-CTAB nanoparticles showed that the IO-CTAB nanoparticles had been very effective in removing As (III) ions from the contaminated aqueous solutions. Thus, for the IO-CTAB nanoparticle samples, an adsorption capacity of 80.841 mg (As)/g, and a K_L_ coefficient value of 0.1 L/mg was obtained. For a better understanding of the mechanisms involved in the removal of arsenic ions from aqueous solutions using IO-CTAB nanoparticles, the Freundlich model was also employed. The Freundlich adsorption isotherm [47] was the first relationship that described a nonideal and reversible adsorption mechanism, having the ability not to be limited to a monolayer formation. Therefore, this empirical model has been applied for multilayer adsorption experiments with an uneven distribution of heat of adsorption and affinity of heterogeneous surfaces [48]. This model was developed for the adsorption of animal-derived coal, demonstrating that the mass adsorbent ratio offered by the adsorbent and solutions is not constant at different concentrations of the solution [49]. From this perspective, the adsorbed amount is the sum of the adsorption in all areas, with the stronger bonding zones being occupied first, and the adsorption energy decreases exponentially at the time of the adsorption process [47].

Figure 3d shows the graphical representations of the (lnQ_e_) function of (lnC_e_) for the As(III) ion adsorption experiments on IO-CTAB nanoparticles. The k_f,_ Freundlich constant is an indicator of the adsorption capacity of the materials used as the adsorbent, while 1/n is a function of the power adsorption from the process [50,51].

The Freundlich model states that if the Freundlich constant n is equal to 1, then the separation of the two phases is independent of the concentration. Moreover, if the value of 1/n is below 1, this is characteristic of a normal adsorption process. Moreover, if the value of 1/n is less than 1, this indicates a cooperative adsorption process [52]. According to the literature, if the value of n is between one and 10, this corresponds to a favorable adsorption process [53]. The values obtained for n from the linearized form of the Freundlich equation for the As(III) ions adsorption experiments on IO-CTAB nanoparticles were greater than 1, leading to a value of 1/n smaller then 1, which corresponds to a normal adsorption process. Table 1 shows the values for both Langmuir and Freundlich constants obtained in the experiments to remove As(III) ions from aqueous solutions using IO-CTAB nanoparticles.

According to previous studies [46,47,54], the separation factor R_L_ indicates the shape of the isotherm. For R_L_ values higher than 1, the isotherm is unfavorable, while for R_L_ = 1, it is linear and for a R_L_ value situated between 0 and 1, it is favorable. In the situation when the R_L_ value is equal to 0, it is irreversible. In this study, the R_L_ values obtained from the experimental data from the batch adsorption experiments, the R_L_ values were between 0 and 1, indicating a favorable adsorption of As(III) on the IO-CTAB nanoparticles. It has also been observed that the uptake of As(III) by IO-CTAB nanoparticles in the batch experiments conducted in this study was high, due to the affinity that is relatively large between As(III) and IO-CTAB nanoparticles. Furthermore, comparing the correlation coefficients (R^2^) obtained from fitting the experimental data using the Langmuir and Freundlich theoretical models, it could be concluded that the experimental equilibrium data of As(III) sorption was best described by the Langmuir model. This behavior may be due to the homogeneous reaction on the surface for As(III) sorption. The results of the batch experiments and the data determined using the Langmuir and Freundlich models emphasized that IO-CTAB nanoparticles were favorable for the adsorption of As(III) ions from aqueous solutions, therefore making IO-CTAB nanoparticles perfect candidates for developing new technologies for water remediation purposes.

### 3.3. Non-Destructive Ultrasound Studies

**Figure 4:**
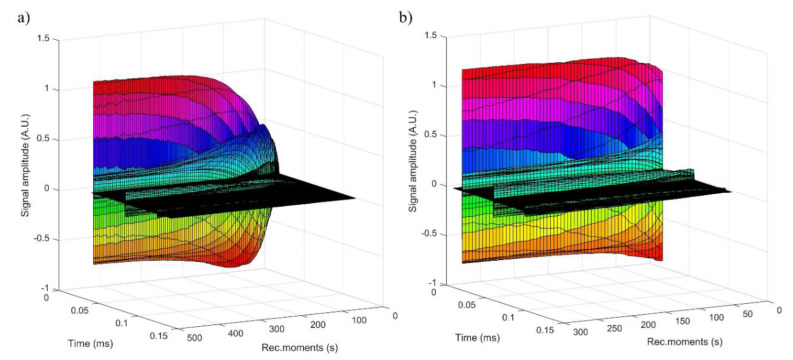
Acquired signals before (**a**) and after (**b**) arsenic removal.

Ultrasound measurements provide information about the interaction with the suspension of solid nanoparticles in the liquid. The amplitudes of the spectral components as well as the velocity of ultrasound propagation in the biphasic system offer important information about the properties of the analyzed sample. In this study, we used ultrasound measurements to highlight the effectiveness of IO-CTAB in removing arsenic from polluted waters, taking double-distilled water as a reference for the measured signals. Figure 4 shows the acquired signals before (a) and after (b) arsenic removal, using two coaxial, identical, longitudinal waves transducers of 5 MHz central frequency, distanced by 30 mm. The time axis represents the duration of the acquisition of an echo in ms. The recording moments axis (Rec. moments) indicates the times at which signal recordings were made, in seconds. A first global information is the evolution during the recordings (Rec. Time) of the maximum level of each echo, related to the level of the corresponding echo of the reference liquid (water), for which the amplitudes remain constant.

**Figure 5:**
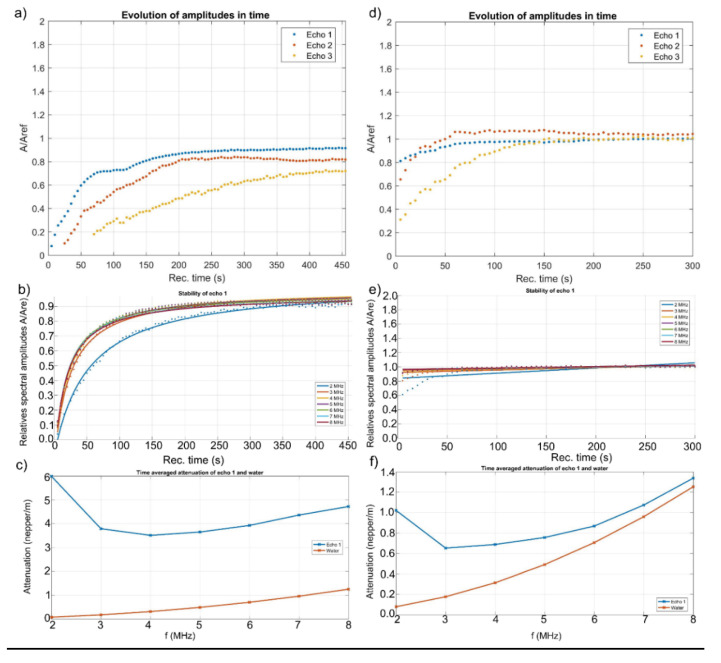
Evolution of amplitude in time before (**a**) and after arsenic removal (**d**); stability of echo 1 before (**b**) and after (**e**) arsenic removal; time averaged attenuation of echo 1 and water before (**c**) and after arsenic removal (**f**).

Each recorded signal was separated into echoes. For each of the three measured echoes, extracted from the 91 signals, was plotted the evolution of the maximum level of the signal depending on the moment of recording. The maximum level was presented relating to the constant amplitude of the respective echo of the reference sample, which was double-distilled water (Figure 5a,d). Considering that the amplitude of the signal through deionized water is of unit value, it is observed that, by decontaminated water (Figure 5d), the relative amplitudes (A/A_ref_) of the three echoes have values equal to those of the reference fluid (deionized water) starting with Rec. time = 100 s. Before decontamination, the relative amplitudes of the three echoes were less than 1, even at Rec. time = 450 s (Figure 5a). Moreover, it was observed that before decontamination, A/A_ref_ of echo 1 at Rec. time = 0.1 s was less than 0.1 (Figure 5a). After decontamination, A/A_ref_ of echo 1 at Rec. time = 0.1 was equal to 0.8 (Figure 5d). This behavior revealed the efficiency of the IO-CTAB biocomposite in removing arsenic from water. The attenuation of each echo depends on time, if the sample is unstable. Both contaminated and decontaminated water were stirred before being measured. The presence of the pollutant leads to an instability of the sample, which can be observed. To follow this evolution, the time dependence of several spectral components (2–8 MHz) was plotted. The time evolution of the relative amplitudes of the spectral components of the first echo are presented (Figure 5b,e). In the case of polluted water, a strong variation of the frequency amplitudes was observed (Figure 5b) while in the case of depolluted water (Figure 5e), the relative amplitude values of the frequencies were around 1 at all times, as for the reference fluid. This behavior is in agreement with that observed in the study of the time evolution of the relative amplitudes of the echoes and confirms the efficiency of IO-CTAB in removing arsenic from water. The efficiency of IO-CTAB in removing arsenic from water is very well highlighted in Figure 5c,f, which shows the time-mediated evolution of the spectral attenuation of echo 1. In arsenic contaminated water, the maximum attenuation at 2 MHz was equal to 8 Np/m (Figure 5c), while after arsenic removal, the maximum attenuation value was equal to 1 Np/m (Figure 5f). On the other hand, it was observed that the minimum attenuation in the case of the polluted sample was at 4 MHz, while after arsenic removal, the minimum attenuation measured was at 3 MHz. Moreover, the time-mediated evolution of the spectral attenuation of echo 1 before the removal of arsenic had much higher values than for double-distilled water (reference fluid). After the removal of arsenic (Figure 5f), the time-mediated evolution of the spectral attenuation of echo 1 followed the time-mediated evolution of the spectral attenuation of the double-distilled water. The stability parameter for the first echo, calculated for the polluted water was equal to 0.418 (1/s), while after the removal of arsenic, it was equal to 0.034 (1/s) compared to that of the double-distilled water (as the reference fluid), which was equal to zero.

### 3.4. X-Ray Photoelectron Spectroscopy Studies (XPS)

**Figure 6:**
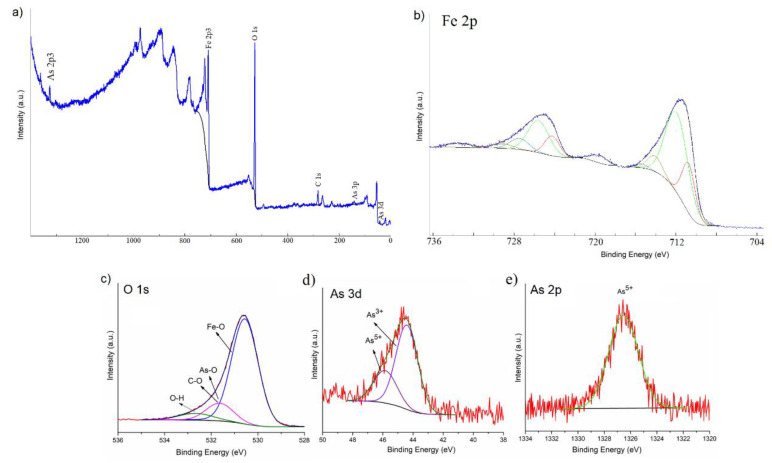
XPS general spectrum of IO-CTAB after As removal (**a**) and deconvolution of Fe 2p (**b**); O 1s (**c**); As 3d (**d**); As 2p (**e**) XPS peaks of the sample after the adsorption of As on IO-CTAB adsorbent.

Figure 6 showed the general XPS scan spectrum of IO-CTAB after arsenic removal from treated water (a) and deconvolution of Fe 2p, O 1s, As 3d, and As 2p XPS peaks. The high resolution XPS spectrum of Fe 2p collected after arsenic adsorption by IO-CTAB are presented in Figure 6b. The peaks at the binding energies of 711.75 eV and 725.59 eV suggest the presence of Fe(II) ion [55]. According to previous studies [56], the peaks at the binding energies of 710.85 eV and 724.24 eV can be assigned to Fe(III). The peaks at the binding energies of 714.11 eV and 727.46 eV could represent an iron compound, of arsenate type, taking into account that we have As with valence (V) in the As spectrum. The peaks at energy binding of 715.37eV and 729.13 eV represent surface structures and shake-up-related satellites of Fe(II). The peaks at energy binding of 719.61 eV and 733.43 eV represent surface structures and shake-up-related satellites of Fe(III).

The high resolution XPS spectrum of O 1s collected after arsenic adsorption by IO-CTAB was composed of three individual components at 532.97, 531.5, and 530.5 eV (Figure 6a).

In agreement with anterior studies [57,58,59], the peak at 530.5 eV was assigned to the Fe–O bonds. According to previous studies [60], the peak at 531.5 eV could be ascribed to As–O and the oxygen components of the carboxyl groups (Figure 6c). The peak at 532.97 was assigned to the surface hydroxyl groups and simple carbon bonds (Figure 6c). The strong affinity between arsenic and IO-CTAB was also confirmed by XPS studies. XPS results confirmed that the As(III) was oxidized to As(IV), as can be seen in Figure 6.

Next, the As 3d and 2p XPS spectra and their deconvolution were presented (Figure 6d,e). The high resolution XPS spectrum of As 3d was deconvoluted into individual component peaks from the arsenic(III) and (V) oxidation states [61]. The high resolution XPS spectrum of As 3d (Figure 6d) was deconvoluted into two individual component peaks that appeared at binding energies (BE) of 44.4 and 45.85 eV for As (III) and As (V) oxidation states, respectively.

These results concerning the deconvolution of the As 3d spectrum were in agreement with the previous studies [61,62]. The high resolution XPS spectrum of As 2p (Figure 6d) revealed a single peak located at the BE of 1326.4 eV assigned to arsenic(V) oxidation states [63]. The high resolution XPS spectra of As2p and As3p revealed that some of the As(III) oxidized to As(V) in the solution or at the IO-CTAB surface. All results revealed that IO-CTAB, on one hand, can remove As(III) from water and on the other hand, it can oxidize As(III) to As(V) simultaneously. Oxidation of As(III) to As(V) leads to a decrease in its toxicity during the adsorption process as As(V) is less toxic than As(III).

### 3.5. Biological Assays

**Figure 7:**
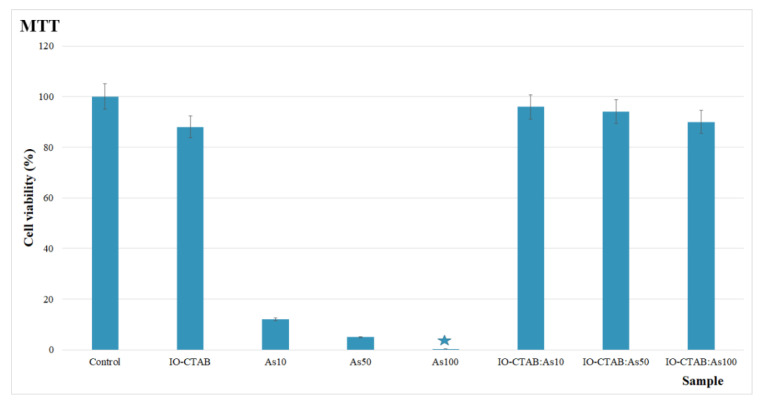
Cell viability of HeLa cells incubated with water in the presence of IO-CTAB nanoparticles, As(III) contaminated solutions, and decontaminated using IO-CTAB nanoparticles. HeLa cell culture was used as the control.

To determine both the biocompatibility of IO-CTAB nanoparticles and the ecotoxicity of the IO-CTAB nanoparticles, in vitro cytotoxicity studies were performed on IO-CTAB nanoparticles, solutions contaminated with various concentrations of As(III) ions, and decontaminated aqueous solutions. The toxicity of contaminated and decontaminated solutions under laboratory conditions was investigated by performing in vitro cytotoxicity studies on HeLa cell cultures. The HeLa cells were also used to determine the biocompatibility of IO-CTAB nanoparticles. The results of the cell viability test of HeLa cultures incubated with solutions contaminated with arsenic ions at different concentrations (As10, As50, and As100) and with decontaminated solutions using IO-CTAB nanoparticles (IO-CTAB:As10, IO-CTAB:As50, and IO-CTAB:As100) are presented in Figure 7. The results of the cell viability of HeLa cultures incubated for 24 h with solutions contaminated with arsenic ions showed that the solutions contaminated with arsenic ions had a high toxicity toward HeLa cells. Moreover, it has been observed that the survival rates of HeLa cells significantly decreased with the increase in the arsenic ions concentration. Thus, for concentrations of 10 mg/L, the cell viability of the HeLa culture was 12%, and for concentrations of 50 mg/L, the cell viability of the HeLa culture was 5%, while for the solution contaminated with a concentration of 100 mg/L, the arsenic ions were 0.3%. A free HeLa cell culture was used as the control. These results are in agreement with data presented by other researchers in studies performed on the toxicity of arsenic ions on different types of cell cultures [64,65,66]. Moreover, the results showed that the toxicity of contaminated solutions can be correlated with the concentration of arsenic ions, showing that cell viability decreased with the increase of the concentration of arsenic ions in contaminated solutions. Furthermore, the results of the cytotoxicity assays of the aqueous decontaminated solutions using IO-CTAB nanoparticles are also presented in Figure 7. The results emphasize that the decontaminated solutions had no effect or a small toxic effect on the cell viability of the HeLa culture after 24 h of incubation. The MTT test showed that for the decontaminated solutions, the cell viability of the HeLa culture was above 90%, which highlighted that the IO-CTAB nanoparticles were efficient in removing the As(III) ions from the aqueous solutions. Moreover, the cytotoxicity of the IO-CTAB nanoparticles was also investigated and the results are depicted in Figure 7. The results of the MTT assay showed that IO-CTAB presented no toxicity on HeLa cells after 24 h.

**Figure 8:**
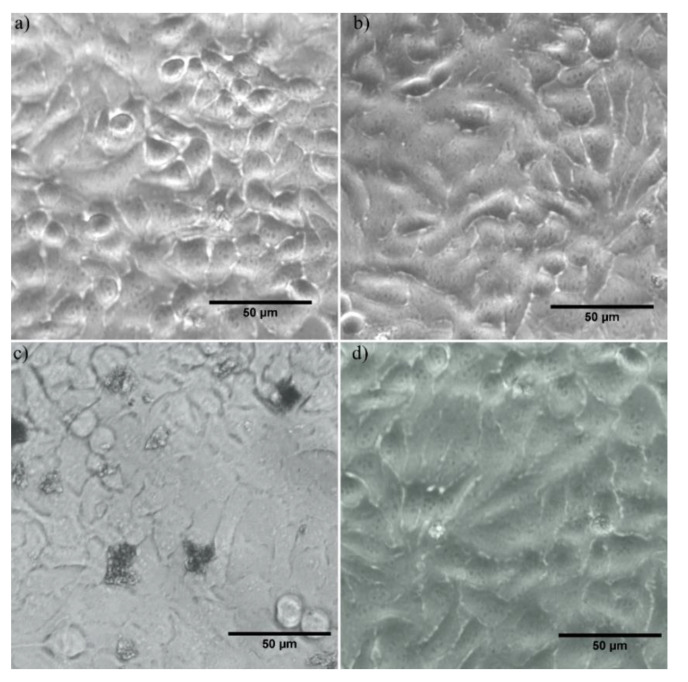
The morphology of the HeLa cells used as the control (**a**); incubated with IO-CTAB nanoparticles in water (**b**), incubated with As(III) contaminated solutions (**c**), and incubated with solutions decontaminated using IO-CTAB nanoparticles (**d**).

Furthermore, the morphology of the HeLa cells incubated with As(III) contaminated solutions with an arsenic concentration of 50 mg/L and decontaminated using IO-CTAB nanoparticles as well as IO-CTAB nanoparticles is depicted in Figure 8.

The results confirmed that the IO-CTAB nanoparticles did not induce any morphological changes in the HeLa cells and also did not affect their proliferation after 24 h of incubation. Moreover, the same results have been obtained in the case of the aqueous solution with 50 mg/L arsenic concentration decontaminated using IO-CTAB nanoparticles (IO-CTAB:As50). On the other hand, the results of the optical microscopy visualization of HeLa cells morphology after being incubated for 24 h with an arsenic contaminated solution (As50) showed that the arsenic ions had a high toxic effect on the HeLa cells morphology and also inhibited their proliferation. These results are in perfect agreement with the results obtained from the MTT quantitative assay and also with previously reported data regarding arsenic toxicity on different types of cells. These preliminary results regarding the cytotoxicity of IO-CTAB nanoparticles demonstrate that they could be successfully used in environmental applications for arsenic removal from aqueous solutions.

## 4. Conclusions

This research demonstrated, for the first time, the effectiveness of IO-CTAB as an adsorbent for the removal and oxidation of As(III) from contaminated water. Iron oxide coated CTAB was synthesized using an adapted co-precipitation method. All evaluation techniques for the removal of arsenic from contaminated water have highlighted the effectiveness of IO-CTAB. Additionally, the XPS analysis showed that during the adsorption process, As(III) can be oxidized simultaneously to As(V), which contributes to the decrease of arsenic toxicity. On the other hand, ultrasound studies have shown a behavior similar to that of double distilled water for depolluted water. The results of the arsenic removal batch experiments revealed that IO-CTAB nanoparticles were favorable for the adsorption of As (III) ions from aqueous solutions. The results also suggest that IO-CTAB nanoparticles could be suitable candidates for the development of new technologies for water treatment purposes. In addition, the cytotoxicity of IO-CTAB nanoparticles was also investigated. The results of the MTT assay revealed that the IO-CTAB nanoparticles did not present any toxic effect against HeLa cells after 24 h of incubation. More than that, the results of the cytotoxic experiments depict that the aqueous solution decontaminated using IO-CTAB nanoparticles did not present toxic effects against HeLa cells and did not induce any changes in their morphology.

## Figures and Tables

**Table 1:** Langmuir and Freundlich isotherm parameters for As(III) adsorption onto IO-CTAB nanoparticles.

Sample	Langmuir				Freundlich		
**IO-CTAB**	R^2^	q_m_ (mg/g)	K_L_ (L/mg)	R_L_	R^2^	n	k_f_
	0.996	80.841	0.1	0.2	0.985	1.06	4.563
